# Screening for Deep Vein Thrombosis Using D-dimer Levels Based on Surgical Patients’ Characteristics

**DOI:** 10.7759/cureus.75565

**Published:** 2024-12-11

**Authors:** Fumimasa Kitamura, Yuta Shiraishi, Kazuya Sakata, Noboru Takata, Kazunori Harada, Ichiro Yoshinaka, Masaaki Iwatsuki

**Affiliations:** 1 Surgical Gastroenterology, Amakusa Regional Medical Center, Amakusa, JPN; 2 Surgical Gastroenterology, Kumamoto University, Kumamoto, JPN

**Keywords:** d-dimer, deep vein thrombosis, pulmonary embolism, screening, surgery

## Abstract

Purpose

Owing to the shortage of surgeons and the decrease in medical staff in regional medical care, reducing unnecessary tests can limit the burden on the staff. In this study, we aimed to examine the predictors of deep vein thrombosis (DVT), such as D-dimer levels in patients who underwent surgery at our hospital, and determine the feasibility of screening in these patients. Knowledge of D-dimer levels can indicate the risk of DVT in patients about to undergo surgery.

Methods

We retrospectively analyzed 310 of 1,059 surgical cases in which preoperative lower extremity ultrasonography was performed in our department between April 2021 and June 2024. We compared 46 patients with thrombi and 264 patients without thrombi.

Results

Patients with low body mass indices (<18.5 kg/m^2^) and high D-dimer levels (> 2 μg/mL) had a significantly higher risk of DVT, whereas patients taking oral antiplatelet drugs or anticoagulants had a significantly lower risk of DVT. The area under the curve for D-dimer levels in predicting DVT was 0.779. D-dimer levels of 2 μg/mL had high sensitivity - (1 - specificity). However, there were three false-negative cases, and the highest D-dimer level that resulted in 100% sensitivity was 1.4 μg/mL.

Conclusion

Predicting DVT using D-dimer levels may be effective, and considering additional testing based on D-dimer levels and patient background may reduce excessive preoperative testing.

## Introduction

Pulmonary embolism (PE) is the most fatal perioperative complication and requires careful attention [1]. Deep vein thrombosis (DVT) is involved in the development of PE, and each facility performs lower-extremity venous ultrasonography and contrast-enhanced computed tomography before surgery to prevent postoperative occurrence [2,3].

However, the incidence of DVT is low, at approximately 0.3% (0.1%-0.7%) [4], and healthy patients undergoing surgery rarely show symptoms suggestive of DVT. Although lower extremity ultrasonography and contrast-enhanced computed tomography (CECT) should be considered for most patients, they cannot be performed in all cases, as they require considerable time and effort.

Currently, D-dimer measurement is used as an indicator for perioperative DVT screening [5]. In cases with high D-dimer levels, DVT is suspected and tests are performed; however, the reference value varies depending on the facility. Owing to the shortage of surgeons and the decrease in medical staff in regional medical care, it is necessary to reduce unnecessary tests as much as possible, which is a problem that concerns surgeons. This study was conducted to examine the predictors of DVT in patients who underwent surgery at our hospital and to determine whether a simple, reliable screening test is possible.

## Materials and methods

Patients and design

**Figure 1:**
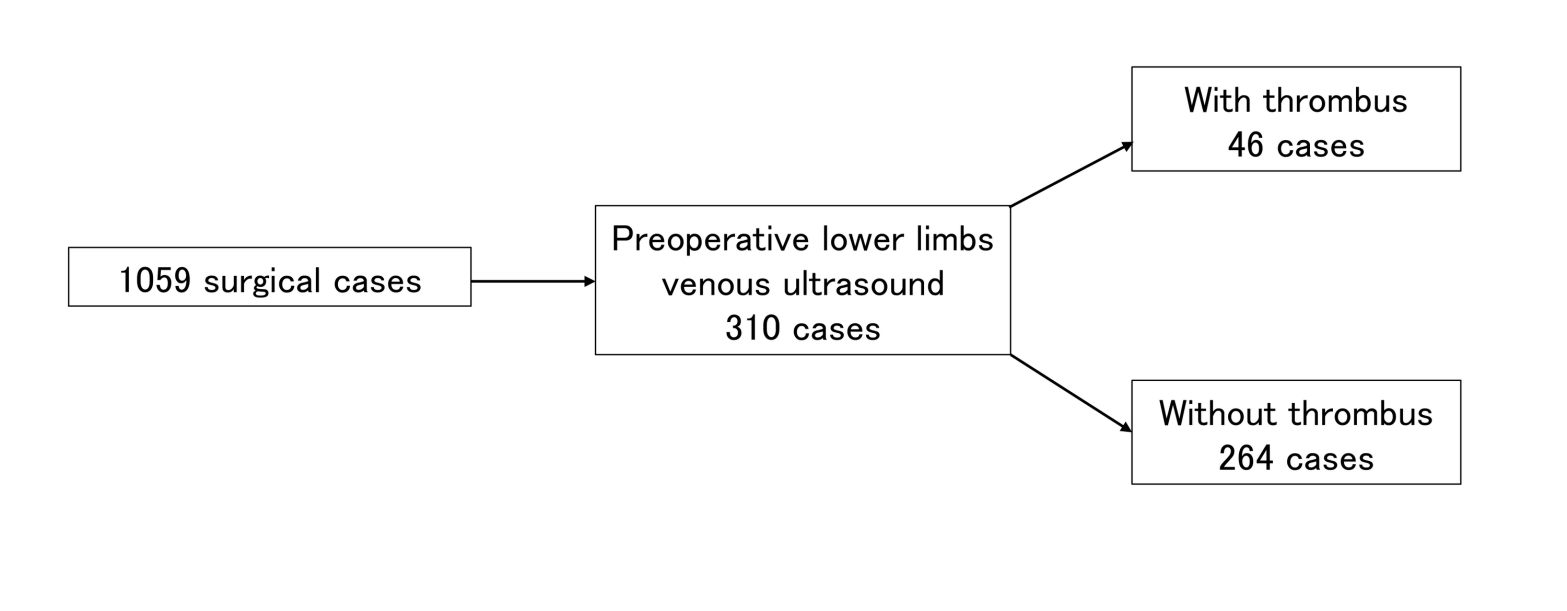
Grouping of patients with lower extremity venous thrombosis using lower extremity ultrasound

At our hospital, lower-extremity ultrasounds are performed at the attending physician’s discretion, especially if there is time before surgery. D-dimer = 2 μg/mL is often used as the standard, but testing may be performed even if the value is low. However, scans are not performed outside of business hours or on holidays. We retrospectively analyzed the data of 1,059 surgical cases where lower-extremity ultrasound was performed at our department. Of these, 310 cases were selected for analysis: 46 patients with thrombi and 264 patients without thrombi (Figure 1).

Data collection

The following patient clinical data were collected from medical records: age, sex, body mass index (BMI), long-term bedridden status, smoking, history of lower extremity orthopedic disease, oral antiplatelet/anticoagulant use, malignant tumors, infections, emergency surgery, nutritional status (preoperative Conut score), D-dimer level, cardiac contractility, and reduced cardiac wall motion.

Evaluation method

Patient data were used to investigate factors related to DVT formation in patients with and without DVT. The predictive value of the D-dimer level for DVT occurrence was determined using receiver-operating characteristic (ROC) curve analysis.

Data analysis

Continuous variables were expressed as means and standard deviations. The chi-square test or Fisher’s exact probability test was used to assess between-group differences in categorical variables, whereas the Wilcoxon test was used for continuous variables. ROC curve analysis was performed to evaluate the area under the curve (AUC) of the D-dimer level for predicting DVT, and the optimal cut-off D-dimer level was determined using Youden’s index. Univariate and multivariate logistic regression analyses were performed to identify predictive risk factors for DVT. Statistical significance was set at p < 0.05. All statistical analyses were performed using JMP pro® version 16 (SAS Institute, Cary, NC, USA).

## Results

Table 1 summarizes the clinical characteristics of the patients. The median age was 80 years, with a slightly higher proportion of women (177 cases, 57%). Thirty-eight patients (12%) were bedridden for over a year. There were 102 cases (32%) of malignant tumors, and 110 patients (35%) underwent emergency surgery. The median D-dimer level was 2.2 μg/ml (Table 1). The most common primary diseases were gallstone or cholecystitis; colon or rectal cancer; and inguinal, femoral, or obturator hernias (Table 2). DVT was observed in 46 of the 310 patients (14.8%) (Figure 1). Patients with thrombosis were treated with prophylaxis on a case-by-case basis. No patient developed clinical PE during the period from surgery to discharge. Univariate analysis of thrombosis factors showed that the risk of thrombosis was significantly higher in older patients (>75 years old) and patients with low BMI (<18.5 kg/m^2^), long-term bed rest, high Conut score (>2), and high D-dimer levels (>2 μg/ml). In contrast, patients with a history of smoking and those taking oral antiplatelet drugs and anticoagulants had a lower risk of thrombosis (Table 3).

**Table 1:** Background of patients who underwent a lower limb ultrasound (n=310) This table shows the overall background of this study. Values ​​are median values. Figures in brackets indicate minimum-maximum values.

Variables	n = 310
Age（year）	80 (28-102)
male/female	133/177
BMI(kg/m2)	21.2 (12.2-37.1)
Long-term bed rest(+/-)	38/272
Smoking(+/-)	123/187
Orthopedic disease of the lower limbs(+/-)	61/249
Antiplatelet drugs(+/-)	43/267
Anticoagulants(+/-)	27/283
Malignant tumors	102/208
Infection	87/223
Emergency surgery	110/200
Conut score	2 (0-9)
D-dimer(μg/ml)	2.2 (0.3-49.2)
EF	68.2 (26.4-87.6)
Decreased cardiac wall motion(+/-)(n=300)	49/251

**Table 2:** Primary diseases of patients who underwent a lower limb ultrasound This table shows the diseases included in this study.

Diseases	n = 310
Gallstones/cholecystitis	73
Colon and rectal cancer	71
Inguinal/femoral/obturator hernia	62
Gastric cancer	21
Peritonitis	16
Appendicitis	12
Strangulation ileus	11
Pancreatic cancer	8
Abdominal wall hernia	7
Others	29

**Table 3:** Examination of thrombogenic factors by univariate or multivariate analysis This table shows univariate and multivariate analyses of thrombosis factors in the thrombosis formation and non-thrombosis formation groups. * indicates a significant p-value (p<0.05)

Factors	Univariate			Multivariate		
	HR	95%CI	P	HR	95%CI	P
Age (<75）	3.50	1.511-8.14	0.0035	2.33	0.84-6.42	0.10
Sex	0.644	0.343-1.20	0.17	-	-	-
BMI(kg/m2)(18.5	2.93	1.48-5.78	0.002*	2.40	1.02-5.69	0.047*
Long-term bed rest	4.37	2.05-9.31	0.0001	2.14	0.85-5.36	0.10
Smoking	0.42	0.20-0.87	0.02	1.18	0.47-2.96	0.71
Orthopedic disease of the lower limbs	0.99	0.45-2.18	0.98	-	-	-
Antiplatelet drugs	0.24	0.05-1.05	0.022*	0.18	0.038-0.88	0.012*
Anticoagulants	1.74e-7	0	0.0025*	9.21e-8	0	0.016*
Malignant tumors	1.53	0.80-2.91	0.80	-	-	-
Infections	1.44	0.74-2.81	0.27	-	-	-
Emergency surgery	1.48	0.78-2.80	0.22	-	-	-
Conut score (2)	2.92	1.48-5.74	0.0014	1.42	0.65-3.08	0.36
D-dimer (2)	14.44	4.37-47.7	0.0001*	14.3	3.24-63.1	<0.0001*
EF (<60)	1.56	0.75-3.25	0.24	-	-	-
Decreased cardiac wall motion	0.63	0.23-1.70	0.349	-	-	-

**Figure 2:**
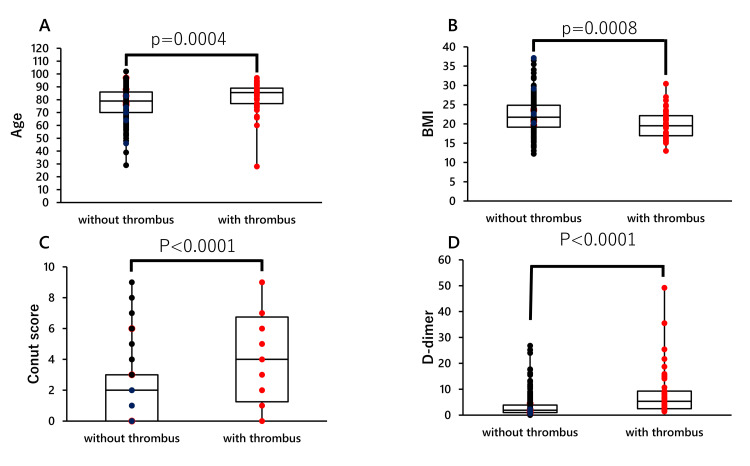
Examination of factors in the formation of lower extremity thrombosis using continuous variables (Wilcoxon analysis) A: Wilcoxon analysis by age and presence of DVT; B: Wilcoxon analysis by BMI and presence of DVT; C Wilcoxon analysis by Conut score and presence of DVT; D: Wilcoxon analysis by D-dimer value and presence of DVT DVT: deep vein thrombosis

Analysis of age, BMI, Conut score, and D-dimer levels showed that patients with DVT were elderly, thin, malnourished (high Conut score), and had high D-dimer levels (Figure 2). Furthermore, multivariate analysis showed that patients with a low BMI (<18.5 kg/m^2^) and high D-dimer levels (>2 μg/ml) had a significantly higher risk (BMI: p=0.047, D-dimer: p<0.0001) of thrombosis, and patients taking oral antiplatelet drugs or anticoagulants had a significantly lower risk of thrombosis (Table 3). The incidence of DVT in the patients taking antiplatelet drugs was 4.7% (2/42). No DVT was observed in patients taking anticoagulants.

**Figure 3:**
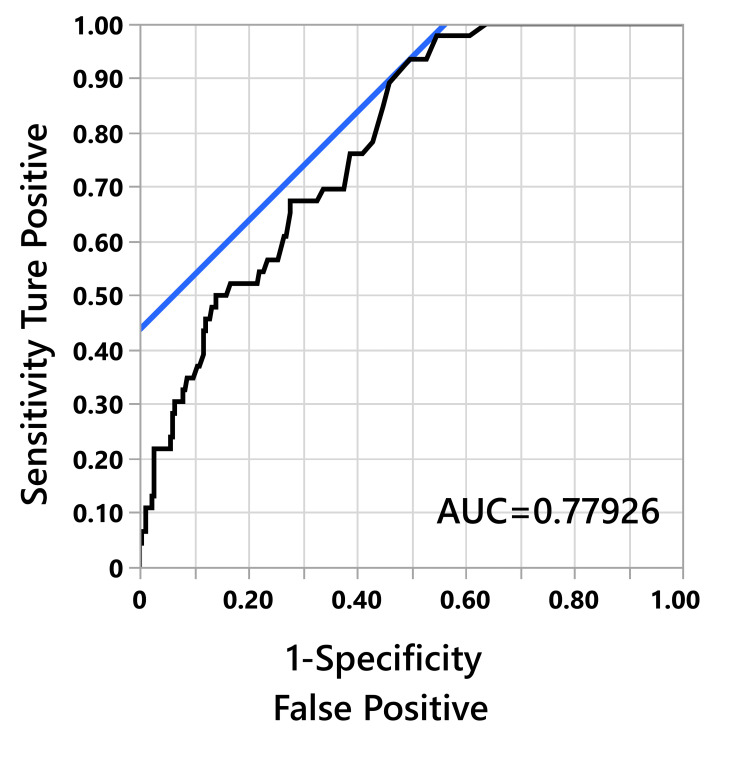
ROC curve of D-dimer and thrombus ROC: receiver-operating characteristic

When the ROC curve for thrombosis screening using D-dimer was created, the AUC was 0.779 (Figure 3), and D-dimer = 2 μg/ml had high sensitivity - (1 - specificity) (Table 4). However, there were three false negative cases, and the highest D-dimer value that resulted in 100% sensitivity was 1.4 μg/ml.

**Table 4:** Details of the ROC curve for D-dimer and thrombus This table shows the sensitivity and specificity of D-dimer in DVT-positive patients. * indicate based on the highest Youden indices of the ROC analysis, the optimal cutoff values of the D-dimer concentration ROC: receiver-operating characteristic; DVT: deep vein thrombosis

D-dimer	1-Spec	Sens	Sens-(1-Spec)	True Posi	Ture Nega	False Posi	False Nega
3	0.3156	0.6739	0.3583	31	180	83	15
2.5	0.3878	0.7609	0.3502	35	161	102	11
2	0.4981	0.9348	0.4367*	43	132	131	3
1.8	0.5475	0.9783	0.4307	45	119	144	1
1.6	0.5894	0.9783	0.3889	45	108	155	1
1.5	0.6084	0.9783	0.3699	45	103	160	1
1.4	0.6388	1	0.3612	46	95	168	0
1.3	0.6540	1	0.3460	46	91	172	0

**Figure 4:**
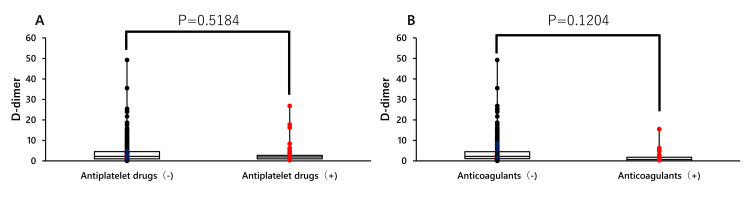
D-dimer levels in antiplatelet and anticoagulant drugs A: Wilcoxon analysis of antiplatelet drug use and D-dimer values; B: Wilcoxon analysis of anticoagulant use and D-dimer values

Finally, the effects of antiplatelet drugs and anticoagulants on the D-dimer levels were investigated. Neither antiplatelet drugs nor anticoagulants had a significant effect on D-dimer levels (Figure 4).

## Discussion

PE is a serious perioperative complication; however, because of its low incidence rate [6], many surgeons have difficulty diagnosing and treating it when it does occur. Therefore, detecting DVT, which is a cause of PE, before surgery is very important; however, there is no standardized test, and screening methods vary from facility to facility. There are screening tests for lower limb thrombosis such as the Wells score [7]. The Wells score is a useful tool when PE is suspected; however, patients suspected of having thrombosis or PE before surgery are rare, and there is currently no time to perform physical examinations in all cases.

D-dimer is widely used as a preoperative predictor of DVT [8,9]. Although the cutoff value varies among facilities, if the threshold is too low, the frequency of lower-limb ultrasound examinations and contrast CT increases. Therefore, it is important to change D-dimer levels of cut-off based on the patient's background.

Previous reports have shown that factors such as older age [10], high BMI [11], prolonged bed rest [12], and malignant tumors [13] lead to thrombosis. However, in this study, low BMI was an independent determinant factor. Patients with obesity are generally considered to be at high risk of DVT. The median age of the patients was 80 years, and in an aging region with a declining population, a decrease in physical activity rather than obesity may be involved [14].

In addition, patients taking antiplatelet drugs or anticoagulants have a lower risk of thrombosis. This may be because anticoagulants are used to treat thrombosis [15]. However, there are reports that antiplatelet drugs are also effective in preventing thrombosis [16]. Furthermore, the use of antiplatelet drugs and anticoagulants may mask D-dimer levels, but no such effect was observed [17,18]. In patients taking these drugs, it may be possible to omit lower limb ultrasonography and contrast CT.

In this study, a D-dimer level of 2 μg/ml may have been highly accurate as we observed only a few false negatives. However, since PE can be fatal, it is important to lower the test threshold in cases where there is a risk of the disease. The threshold value for D-dimers has been discussed in various papers; most studies obtained D-dimer levels of approximately 1-2 μg/ml, which is a reasonable range in this study [19,20]. Using a D-dimer level of 2 μg/ml as the standard, in cases where the risk of thrombosis is high, additional testing should be performed if D-dimer levels are 1.4 μg/ml or higher, and in patients taking antiplatelet drugs or anticoagulants, it is thought that testing may be omitted regardless of D-dimer levels. As shown in this study, by considering D-dimer levels and patient background, it may be possible to omit lower-limb ultrasonography. In addition, this study excluded common risk factors that are rare such as estrogen administration [21], a history of varicose veins [22], chemotherapy [23], and a predisposition to thrombosis [24,25]. However, if such a background is present, thrombosis screening should be considered.

This study has two major limitations. First, this is a small-scale, single-center, retrospective cohort study, with potential study bias; hence, future large-scale prospective studies are needed. Second, DVT was evaluated using only ultrasonography. However, the diagnostic accuracy of DVT may be affected by ultrasonography alone. Therefore, future studies should use additional modalities for accurate preoperative DVT evaluation.

## Conclusions

Predicting DVT using D-dimer levels may be effective, and by considering additional testing based on D-dimer values and patient backgrounds, excessive preoperative testing may be reduced. Establishing a consistent background value for D-dimer values in perioperative patients is important for future success.
